# INTEGRATING HOME HEALTH IN AN INTEGRATED CARE SYSTEM: VETERAN EXPERIENCES WITH A VA PILOT PROGRAM

**DOI:** 10.1093/geroni/igad104.3436

**Published:** 2023-12-21

**Authors:** Heather Davila, Mary Good, Aaron Seaman, Tammy Walkner, Irene San Roman, Heather Benzel, Samantha solimeo

**Affiliations:** Iowa City VA Health Care System, Iowa City, Iowa, United States; Iowa City VA Health Care System, Iowa City, Iowa, United States; University of Iowa, Iowa City, Iowa, United States; Iowa City VA Health Care System, Iowa City, Iowa, United States; Iowa City VA Health Care System, Iowa City, Iowa, United States; VA Midwest Healthcare Network (VISN 23.), Grand Island, Nebraska, United States; Iowa City VA Health Care System, Iowa City, Iowa, United States

## Abstract

The Veterans Health Administration (VA) serves nearly 3 million Veterans aged 65 and over, 350,000 of whom use skilled home health (e.g., nursing, rehabilitation therapy) or homemaker/home health aide services annually. Despite being the nation’s largest integrated healthcare system, VA has traditionally purchased home health services from contract agencies. Coordinating care between VA and contract agencies is an ongoing challenge, and accessing home health has become increasingly difficult, especially in rural areas. To address these challenges, the Iowa City VA launched VA’s first “VA Home Health” (VAHH) program in 2021. In 2022, the VA Midwest Health Care Network (VISN 23) expanded VAHH to three additional sites, with over 700 Veterans served to date. We interviewed Veterans receiving VAHH who had previously used contract services (n=30) to learn about their care experiences. Veterans described multiple benefits of VAHH over contract services: staff’s access to VA electronic health records, mechanisms for quickly sharing information with other care providers, understanding of Veterans’ unique care needs (e.g., PTSD), and knowledge of the breadth of services VA offers. Most Veterans said they were highly satisfied with VAHH, with many expressing a desire for VAHH to expand and serve more Veterans. Our preliminary findings suggest VAHH may improve Veterans’ experiences with care and overall quality of care. Given VA’s focus on expanding Veterans’ access to home-based services, these preliminary findings could inform national VA decisions surrounding home health delivery. Our findings are also relevant to other healthcare systems as they address similar coordination and access challenges.

